# Comparative Proteomics of Milk Fat Globule Membrane (MFGM) Proteome across Species and Lactation Stages and the Potentials of MFGM Fractions in Infant Formula Preparation

**DOI:** 10.3390/foods9091251

**Published:** 2020-09-07

**Authors:** Michele Manoni, Chiara Di Lorenzo, Matteo Ottoboni, Marco Tretola, Luciano Pinotti

**Affiliations:** 1 Department of Health, Animal Science and Food Safety, VESPA, University of Milan, 20134 Milan, Italy; michele.manoni@unimi.it (M.M.); matteo.ottoboni@unimi.it (M.O.); 2 Department of Pharmacological and Biomolecular Sciences, University of Milan, 20133 Milan, Italy; chiara.dilorenzo@unimi.it; 3 Agroscope, Institute for Livestock Sciences, 1725 Posieux, Switzerland; marco.tretola@agroscope.admin.ch; 4 CRC I-WE (Coordinating Research Centre: Innovation for Well-Being and Environment), University of Milan, 20134 Milan, Italy

**Keywords:** milk fat globules, bovine milk proteins, milk fat globule membrane, comparative proteomics, infant formula preparation

## Abstract

Milk is a lipid-in-water emulsion with a primary role in the nutrition of newborns. Milk fat globules (MFGs) are a mixture of proteins and lipids with nutraceutical properties related to the milk fat globule membrane (MFGM), which protects them, thus preventing their coalescence. Human and bovine MFGM proteomes have been extensively characterized in terms of their formation, maturation, and composition. Here, we review the most recent comparative proteomic analyses of MFGM proteome, above all from humans and bovines, but also from other species. The major MFGM proteins are found in all the MFGM proteomes of the different species, although there are variations in protein expression levels and molecular functions across species and lactation stages. Given the similarities between the human and bovine MFGM and the bioactive properties of MFGM components, several attempts have been made to supplement infant formulas (IFs), mainly with polar lipid fractions of bovine MFGM and to a lesser extent with protein fractions. The aim is thus to narrow the gap between human breast milk and cow-based IFs. Despite the few attempts made to date, supplementation with MFGM proteins seems promising as MFGM lipid supplementation. A deeper understanding of MFGM proteomes should lead to better results.

## 1. Introduction

Bovine milk is an oil-in-water emulsion and is rich in nutrients and bioactive factors. Its unique composition makes it essential for the correct growth and development of newborns [1]. The main milk components are water, fat, proteins (casein micelles and serum proteins such as α-lactalbumin, β-lactoglobulin, blood serum albumin, lactoferrin, enzymes, and immunoglobulins), lactose, and minerals [2,3]. Milk fat occurs as milk fat globules (MFGs) in the water, with a size ranging from 0.1 to 15 μm. MFGs are composed of a nonpolar triglyceride (TG) core and are covered in a layer of surface-active material, which is needed to maintain their stability in the emulsion and to protect them from enzymatic degradation and coalescence. This membrane is called the milk fat globule membrane (MFGM), of which the bovine form is the most studied and employed in the dairy industry [1,3,4,5,6]. Table 1 showcases the MFGM content in the main dairy products, such as milk, cream, and cheese.

Bovine MFGM is about 10–20 nm in cross-section and its mass accounts for 2–6% of the total MFG mass [9]. It is made up of many different compounds: polar lipids such as phospholipids, sphingolipids, and glycolipids, and also cholesterol, proteins, and surface glycoproteins [10]. This membrane acts as a natural emulsifier and encases the nonpolar triglyceride core of MFGs [11,12]. The complex MFGM architecture ensures stable dispersion of MFGs in milk—polar lipids and glycoproteins present in the membrane induce electrostatic and steric repulsion, preventing coalescence and aggregation of the fat globules [3,7,13]. There are several health-promoting effects of the MFGM (mainly from bovine but also from other species), such as anticarcinogenic, antimicrobial, anti-inflammatory, and anticholesterolemic activities [6,7].

The anticarcinogenic activity was assessed on HT-29 cells (a human colon cancer cell line) by three studies [14,15,16], which showed that MFGM could reduce the proliferation and enhance apoptosis of the cancer cells through the activation of effector caspase-3. The antimicrobial activity was observed through the inhibition of in vitro rotavirus infectivity [17] and the anti-adhesive activity exerted by a mucin 1 (MUC1)-enriched MFGM fraction against bacteria in the gut mucosa [18]. The anti-inflammatory activity was evaluated with the in vivo mitigation of lipopolysaccharide (LPS)-induced intestinal damage and inflammation in low birth weight (LBW) mice [19] and with the decrease of pro-inflammatory serum markers such as total cholesterol, low density lipoprotein (LDL)-cholesterol, along with an increased production of anti-inflammatory cytokines in obese adults challenged with a high-fat meal rich in saturated fatty acids [20]. Finally, the anticholesterolemic activity was assessed by the decrease exerted by MFGM-derived sphingomyelin of the intestinal absorption of cholesterol and fats in animal models, thus protecting the liver from fat- and cholesterol-induced steatosis and consequently preventing the inflammatory condition involved in atherosclerosis and insulin resistance [7,21]. To summarize, the MFGM could play a key nutraceutical role in many adverse health conditions, even though its effectiveness and potential claims need to be addressed properly.

The aim of this review is to provide a general overview about the formation of bovine MFGs and MFGM and to highlight the main similarities and differences across the MFGM proteomes of the most-studied species (human, cow, goat, buffalo, etc.) through the analysis of comparative proteomic studies. Moreover, the potential supplementation of MFGM fractions in infant formula (IF) is investigated in order to underline the beneficial effects exerted by MFGM bioactive components in infant feeding.

## 2. Bovine MFGs and the MFGM

### 2.1. Formation of Bovine MFGs and MFGM

The various classes of fatty acids (FAs) of milk fat derive above all from feed and rumen microbial activity. In particular, short-chain fatty acids (SCFAs) and medium-chain fatty acids (MCFAs) derive from de novo synthesis in the mammary gland, involving acetyl-coenzyme A carboxylase (ACC) and fatty acid synthase (FAS) enzymes, starting from acetate and butyrate [22,23]. These two molecules are produced in the rumen by fermentation of feed components, such as carbohydrates. Long-chain fatty acids (LCFAs) generally derive from dietary lipids or mobilization of body reserves and specifically they are released by lipoprotein lipase from TGs or from very low density lipoproteins (VLDL), or further from non-esterified fatty acids (NEFA), which are usually found in plasma bound to albumin [24,25,26]. Once they are synthesized, FAs pass through the basal plasma membrane of mammary gland epithelial cells via diffusion and reach the endoplasmatic reticulum (ER), where the TG droplets are synthesized starting from FA precursors. The microlipid droplets are then extruded from the ER in the cytoplasm—during the extrusion process, the droplets are encased in a surface-active inner monolayer, which surrounds the TG core and is made up of polar lipids and specific surface-associated proteins derived from the ER [3,27,28].

**Figure 1:**
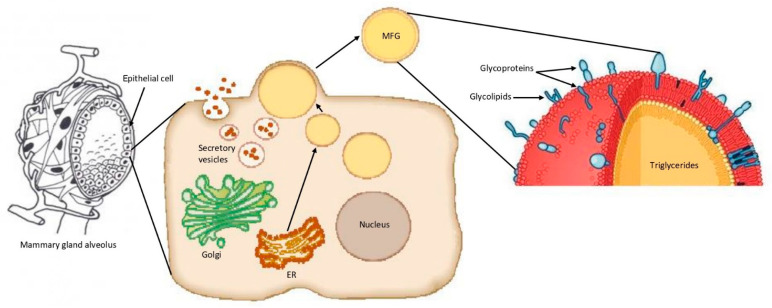
Schematic representation of milk fat globule (MFG) formation. The microlipid droplets (yellow circles) are extruded from the endoplasmatic reticulum (ER) in the cytoplasm of the mammary gland epithelial cell to reach the apical plasma membrane, where they are extruded in the alveolar lumen as MFGs. Adapted from Reece, 2004 [32]; Horseman et al., 2014 [33]; and Wikipedia [34].

Once in the cytoplasm of the mammary gland epithelial cells, microlipid droplets grow in volume and then migrate through the cell cytoplasm, from the basal to the apical pole of the cell. The lipid droplets are secreted by the cells in an apocrine-like mechanism into the alveolar lumen as MFGs, surrounded by the apical plasma membrane of the cells [29,30]. The result of this process is that the MFGM is a trilayer membrane, with the inner layer composed of proteins and polar lipids from the ER, and the outer bilayer of proteins and polar lipids from the apical plasma membrane of the mammary gland epithelial cells (Figure 1) [4,5,28].

In the external layer of the MFGM there are partially embedded, loosely attached and transmembrane proteins, as well as cholesterol molecules associated with polar lipids, while glycoproteins are also present on the surface, with carbohydrate domains oriented outwards [3]. The most widely accepted model for this type of membrane is thus the fluid mosaic model [4,7]. This is because the bovine apical plasma membrane of epithelial secretory cells and the bovine MFGM membrane show a similar distribution of all their components [31].

### 2.2. Lipids in MFG and MFGM, and the Role of Choline

MFG core is predominantly composed of non-polar lipids, named TGs, accounting for 98% of total milk fat. Milk fat is composed of over 400 different FAs, of which 15 represent 90% of the total FA pool. Saturated FAs in bovine milk fat account for 70% of the total milk FAs, with the main forms being palmitic acid (26–32%), stearic acid (12%), and myristic acid (10%). Of the saturated FAs, MCFA (6:0–12:0) represent about 10% of total milk FAs, whereas SCFA (4:0) account for less than 3%. Mono-unsaturated FAs account for 25% of the total milk FAs, with the main form being oleic acid (20–25%). Poly-unsaturated fatty acids (PUFAs) constitute 2.5% of the total milk FAs, and the two major PUFAs are linoleic acid (1–3%) and α-linolenic acid (0.5–2%) [26]. The latter two are among the most important essential fatty acids, which exert an anti-inflammatory function and are also important to prevent cardiovascular diseases in humans [35]. Bovine MFGM is composed mainly of polar lipids that account for 0.2–1% of the total milk fat. The amount of polar lipids in milk fat is related to the amount of the MFGM and then to the size of fat globules [28,36]. The major polar lipids that build bovine MFGM are membrane glycerophospholipids, a group that include phosphatidylcholine (PC, 35–36%); phosphatidylethanolamine (PE, 27–30%); phosphatidylinositol (PI, 5–11%); phosphatidylserine (PS, 3%); and sphingolipids, in particular, sphingomyelin (SM, 25%) [3,7,29,30]. Most of these (about 60%) are choline-containing phospholipids, namely, PC, lysophospatidylcholine (lyso-PC), and sphingomyelin [37,38], showcasing why MFGM constitutes the major choline-containing component of bovine milk [37]. The amount of choline-containing phospholipids in bovine milk is about 105–210 mg/L, to which free choline should be added [38,39,40]. This value is calculated considering that 60% of milk phospholipids contain choline and, furthermore, that phospholipids account for 0.2–1% of total milk lipids [28,32,37,41]. Usually, choline liver reserves and also its metabolites are employed to maintain certain levels of choline secretion into milk. In 1995, Zeisel and collaborators [42] observed that rats fed with a choline-deficient diet had about 90% lower hepatic PC compared to rats fed with a choline-adequate diet. Moreover, lactating rats fed with a choline-deficient diet showed sevenfold higher levels of hepatic TG than non-mated females fed with a choline-adequate diet, whereas TG levels were fourfold higher in lactating rats fed with a choline-adequate diet [42]. According to Kinsella [43], a bovine mammary gland normally yielding 25 L of milk secretes 10 ± 3 g phospholipids per day. This quantity corresponds on average to the 5% of the total phospholipid content of the mammary tissue [43]. These findings confer to choline supplementation an important metabolic role in lipid transport to and within extra-hepatic tissues, such as the mammary gland [44,45,46,47,48].

This idea was confirmed more recently by Li and collaborators also [49]. In particular, the authors evaluated the effects of choline supplementation in intrauterine growth-restricted (IUGR) pigs compared to a normal-choline diet. Choline supplementation decreased hepatic free FAs and TG level, downregulated lipogenic enzyme expression, and enhanced TG export from liver, acting also on cholesterol regulation through higher high-density lipoprotein cholesterol (HDL-C) and lower total plasma cholesterol. Hence, choline supplementation improved hepatic lipid metabolism, avoiding the abnormal lipid metabolism condition of IUGR pigs [49].

Other studies confirmed the importance of choline supplementation in increasing not only milk production (yield and composition) but also choline-containing compounds in milk derived from dairy ruminants [12,50,51,52,53,54,55].

The potential of designing milk with a higher content of choline-containing compounds via choline supplementation in animal feeding is interesting from several points of view. Interestingly, there is scientific evidence about the nutraceutical benefits of phospholipids, sphingolipids, and SM-derived metabolites (ceramide and sphingosine), which have shown antiproliferative activity on cancer cells—MFGM digestion occurs along the entire length of gastrointestinal (GI) tract, with high levels of ceramide and sphingosine recovered in the small intestine and the colon, where they can directly exert their beneficial effects or where they can enter the bloodstream to reach peripheral organs. Indeed, ceramide and sphingosine are two metabolites acting as second messengers in cell signaling, exerting pro-apoptotic and antimitogenic effects [6,14,15].

### 2.3. Major MFGM Proteins

**Figure 2:**
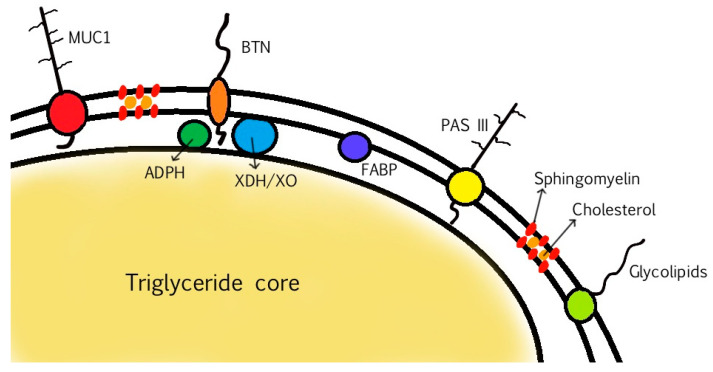
Structure of MFGM and localization of the main MFGM proteins.

MFGM proteins account for 25–60% of the mass of the MFGM, 1–4% of total milk proteins, and 1% of the total globule mass. The proteins can be classified into integral proteins and peripheral proteins, whereas others are partially embedded or loosely attached to the membrane. During the secretion of the MFGs, the constituents are re-arranged within the apical plasma membrane and the MFGM [4,31]. The localization of the proteins thus varies—some are associated with the inner monolayer membrane, while others are associated with the outer bilayer membrane [7,28,29]. The main MFGM proteins are adipophilin (ADPH), butyrophilin (BTN), mucin 1 (MUC1), xanthine dehydrogenase/oxidase (XDH/XO), CD36, periodic acid Schiff III (PAS III), PAS 6/7, lactadherin, and fatty acid-binding protein (FABP), as is shown in Figure 2 [7,56,57].

ADPH, also known as perilipin 2, is a major constituent of the MFGM and is localized in the inner monolayer membrane. It regulates lipolysis by controlling the access of proteins to the MFG [29,56]. BTN is a transmembrane protein and is the most abundant protein in bovine MFGM. BTNs are members of the immunoglobulin (Ig) superfamily, and BTN1A1 is the form in human MFGM [28,29,56]. It has been observed that the knockout of BTN1A1 in bovine mammary epithelial cells decreased the size and the phospholipid content of lipid droplets (the precursors of MFGs), thus suggesting that BTN1A1 has a key role in regulating the synthesis of lipid droplets via a mechanism involving membrane phospholipid composition [36]. MUC1 is a glycoprotein with highly glycosylated extracellular domains localized on the outer surface of MFGs. This feature makes it resistant to digestion and potentially available to act as a decoy receptor for pathogens [18,29,56].

XDH/XO is a redox enzyme that accounts for 12% of total bovine MFGM proteins and is localized in the intermembrane space between the monolayer and the bilayer, forming a tripartite structure with BTN and ADPH (needed to interconnect the inner and outer membrane). It plays a role in antimicrobial defense of the GI tract through the production of reactive oxygen species (ROS) as well as reactive nitrogen species (RNS), which have bactericidal properties. Surface carbohydrates and XDH/XO may possibly act as decoys—pathogens can interact with receptors of the epithelial cells of the GI tract, but can also bind to similar receptors on the MFGM that themselves can act as decoys, such as MUC1 [18], to avoid bacterial interaction with their primary target (GI epithelial cells) and that can also impart an antimicrobial effect thanks to ROS/RNS production by XDH/XO [7,28,29]. Finally, FABP is a protein with a similar localization of XDH/XO and plays a key role in the synthesis of MFG lipid constituents during the intracellular transport of FAs. Indeed, it is involved in the transport of FAs through the capillary endothelium to reach the cytoplasm of mammary endothelial cells, where they cross the membrane via diffusion [24,29].

Interestingly, some authors [7,58] have demonstrated the presence of the onco-suppressors breast related cancer antigens 1/2 (BRCA1 and BRCA2) in human and bovine MFGM. These two onco-suppressors are involved in DNA repair processes [7]. The reason for their presence in the MFGM could be because MFGs are secreted by mammary gland epithelial cells and carry a fraction of their apical plasma membrane. This hypothesis could also explain the presence of these two proteins in human MFGs in secreted milk. In 2002, Vissac and collaborators evaluated BRCA1/2 expression in MFGs of women just after delivery, observing similar patterns of expression of the two proteins [58]. The nutraceutical role of the MFGM and its potential anticancer effect could be explained by the fact that after MFGM consumption, the inhibitory peptides might be released from MFGM and subsequently absorbed by the digestive tract. The absorbed peptides could enter the bloodstream and reach the organs (or tissues), where they inhibit the transforming cells [6].

In Table 2, the major components of bovine MFGM and their main functions are listed.

## 3. Comparison of MFGM Proteome between Different Species and Lactation Stages

Bovine milk is the major substitute for human milk and the most produced animal milk in the world [59]. Bovine MFGM is thus the most studied and employed in the industry of dairy products, for example in the production of IFs [60,61,62]. Milk differs from species to species above all in terms of the composition and the amount of macromolecules. This review discusses the variations in MFGM across species.

**Figure 3:**
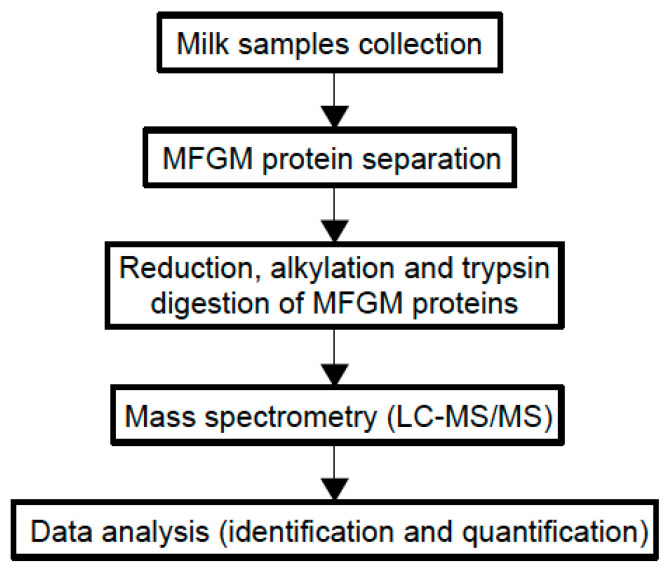
Experimental workflow of MFGM proteome analysis using a proteomic approach.

Although most of the beneficial effects of MFGM are associated with its polar lipid fraction [6], studies on MFGM proteome are increasing since MFGM proteins show bioactive properties and new technologies have enabled more detailed analyses of the MFGM proteome. Comparative proteomic analyses have been performed to understand how proteomes vary between different species and between different stages of lactation [63]. Figure 3 shows the general experimental workflow.

These analyses have been performed to study and compare the proteome of various MFGMs, mainly from humans and cows [64,65,66], but also from other species such as the goat, yak, buffalo, horse, and donkey [61,62,67,68,69,70].

Human milk proteome varies across colostrum and mature milk [71], and these variations are also reflected in the MFGM proteome that varies between lactation stages [72].

This pattern of variations is also valid for other species such as cows [68,73] and goats [70]. In particular, Reinhardt and Lippolis [73] observed that the proteins associated with lipid transport synthesis and secretion (such as FABP) and MUC1 were highly upregulated in 7-day-old milk MFGM than in colostrum MFGM from cow’s milk. The variation of expression of proteins such as FABP is indicative of an early developmental shift in milk fat transport, despite higher fat content in colostrum [73].

In a proteomic study focused on human colostrum MFGM, 107 proteins were identified, half of which were typical MFGM proteins, such as lactadherin and BTN [64]. A similar number of proteins (120) were detected in bovine MFGM. As with human MFGM, BTN was identified as the major MFGM protein, while membrane/protein trafficking proteins (23%) and cell signaling proteins (23%) accounted for almost half of the proteins identified [65].

**Figure 4:**
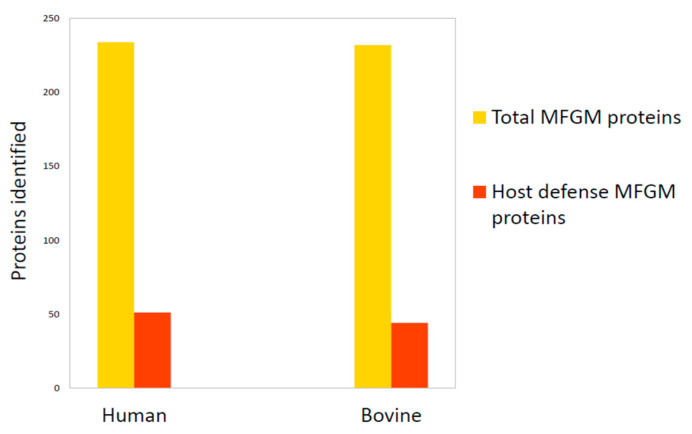
Comparison of total MFGM proteome and host defense MFGM proteins between humans and bovines. Data are from Hettinga et al., 2011 [66].

However, apart from some common features, human and bovine MFGM are different in terms of the fraction of proteome involved in host defense. In 2011, Hettinga and collaborators [66] verified that the total number of host defense MFGM proteins was similar between humans (51 out of 234 proteins identified in human MFGM) and bovines (44 out of 232 proteins identified in bovine MFGM) (Figure 4). However, the human MFGM was more enriched with Igs than bovine MFGM, while bovine MFGM was more enriched with antibacterial proteins. This important information helped identify the main proteins with immunity-promoting properties for newborns [66].

Human MFGM phosphoproteome has recently been studied [72]. Phosphorylation is a post-translational modification that plays a key role in regulating many signaling pathways. The authors identified 203 phosphoproteins, of which 48 were differentially phosphorylated in colostrum and mature milk. These 48 phosphoproteins were mainly associated with immune-related processes. The results showed that there were more immune system process-related phosphoproteins in human colostrum MFGM than in mature MFGM, probably because of the important role that colostrum has in building the immune system of newborns [72].

**Figure 5:**
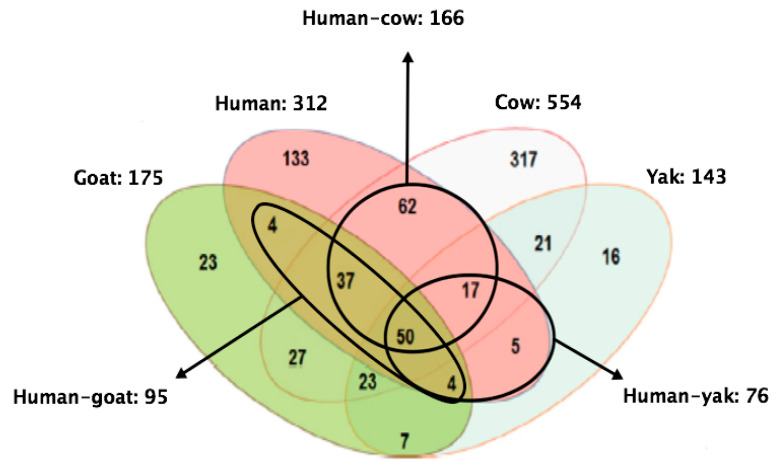
Shared and uniquely identified and quantified proteins in humans, cows, goats, and yaks. Adapted from Lu et al., 2016 [61].

In terms of the MFGM proteome across different species, Lu and collaborators [61] identified and quantified 312, 554, 175, and 143 proteins in human, cow, goat, and yak MFGM, respectively. Fifty proteins were shared among species. Human MFGM shared the highest number of proteins with cow MFGM, whereas with goat and yak MFGM, the number was lower (Figure 5). In terms of composition, the correlation between cow and human MFGM was higher than that between goat and human MFGM, and also between yak and human MFGM. Analyses of the molecular function of proteins revealed that human MFGM was enriched in ER proteins, whereas cow MFGM was enriched in plasma membrane proteins [61]. These findings confirm that MFGM originates from the ER and the plasma membrane [31].

The most shared proteins across species were involved in protein/vesicle-mediated transport, along with major MFGM proteins such as BTN, ADPH, FABP, and MUC1. The main difference regarding human MFGM proteome was a higher enrichment in enzymes involved in lipid catabolism, also reported in Liao et al. [68] and in a set of immune response proteins [61,72].

In 2015, another research team compared the proteome of cow, yak, buffalo, goat, and human MFGM [74]. The authors identified a total of 520 proteins of all species, most of which were shared among all species, although in different isoforms, as also reported by other studies [75,76], such as BTN, lactadherin, MUC1, and ADPH. These findings showed that the MFGM proteome presents a high complexity and variability among species. In terms of molecular function and Gene Ontology (GO) categories, cellular process, localization, transport, signal transduction, and response to stimulus were enriched in all the MFGM fractions [74].

Comparative proteomic analyses have been performed to compare cow and goat proteome. In a 2019 study, a total of 776 MFGM proteins were identified: 427 and 183 that are unique for goat and cow milk, respectively, and 166 proteins shared between the two species. Most of the goat MFGM proteins were related to metabolic processes (about 21%), whereas most of the cow MFGM proteins were related to disease-associated pathways (about 49%) [62]. Subsequently, the same authors evaluated the variations between goat colostrum and mature MFGM proteome. They found a higher number of proteins than in their previous study; in particular, 543 and 858 proteins in colostrum and mature milk, respectively, of which 394 are shared in colostrum and mature milk. Colostrum was found to have fewer proteins but more functions of protein processing in the ER than mature milk, whereas mature milk had more metabolism-related proteins [70].

**Figure 6:**
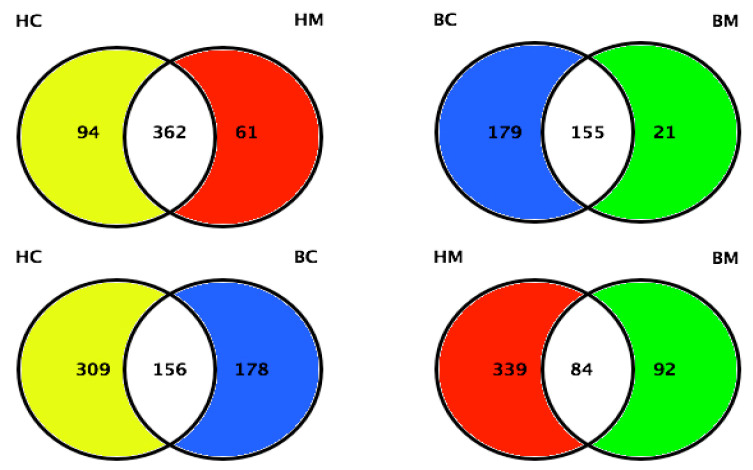
Quantitative comparison of MFGM proteins between human colostrum and human mature milk, bovine colostrum and bovine mature milk, human colostrum and bovine colostrum, and human mature milk and bovine mature milk (HC = human colostrum; HM = human mature milk; BC = bovine colostrum; BM = bovine mature milk). Adapted from Cao et al., 2019 [68].

Along with the analyses performed on proteome and phosphoproteome, the MFGM glycoproteome has also been investigated [67,68]. Cao and collaborators identified and quantified 465, 423, 334, and 176 glycoproteins in human colostrum and mature milk, and bovine colostrum and mature milk, respectively. Human colostrum and mature milk shared 362 glycoproteins, whereas bovine colostrum and mature milk shared 155 glycoproteins (Figure 6). The authors found 24.3% (156) of glycoproteins were shared between human and bovine colostrum, and 16.3% (84) of glycoproteins were shared between human and bovine mature milk. These results indicated more dramatic variations in MFGM glycosylation within species than lactation stages [68].

In another study, Yang et al. investigated the variation among species by analyzing MFGM glycoproteome from cow, buffalo, yak, goat, and human milk. They found that the glycoproteins from the different MFGM species were mainly related to the response to stimulus, according to the GO categories, and that the fractions from ruminants (cow, buffalo, yak, goat) were more similar to each other when compared to the non-ruminant’s fraction (human) [67,74].

An example of the application of these comparative studies was given recently by Ji and collaborators [16], who evaluated the antiproliferative effect of five MFGM fractions from yak, bovine, goat, camel, and buffalo milk using the HT-29 cell line. The antiproliferative effect was evaluated in terms of cell viability, cell cycle, cytomorphology, apoptosis, and mitochondrial membrane potential (MMP). The results showed that all the five MFGM fractions reduced cell growth by affecting cell cycle and inducing apoptosis, whereas MMP values were also significantly reduced by all the five MFGM fractions. Among all the tested samples, buffalo and goat MFGMs were more effective in inducing apoptosis than the other three MFGMs. These data suggest that MFGM might be a putative agent for the prevention of human colon cancer [16].

To summarize, the principal MFGM proteins have been identified in all species. However, the main molecular functions exerted by MFGM proteomes vary according to species and lactation stage due to the variations in protein expression. For example, the human MFGM proteome (in particular that contained in colostrum) has more immune response-related proteins than the MFGM proteome from other species. There are similarities between human and cow MFGM proteome and molecular functions, suggesting that bovine milk, and more specifically bovine MFGM proteins, could be used as a supplement in IFs [77,78].

The varying number of proteins identified and quantified in different studies depends on the proteomic methods performed by the authors. In any case, each study is a step forward in terms of the knowledge regarding MFGM proteome. The potential of these results could facilitate the correct management of the MFGM proteome in the design of products such as IFs supplemented with specific MFGM proteins.

## 4. MFGM: Potentials in Infant Formula Preparation

Breast milk is considered the gold standard for infant nutrition and is required for optimal infant growth, brain and GI tract development, as well as establishing the immune system. Some of the bioactive factors of breast milk are present in the MFGM. In order to develop products that reflect the complexity of human milk, efforts have been made to imitate the nutritional profile and composition of human breast milk. IF has been designed in order to be a suitable alternative to human breast milk [21,77,78,79]. Bovine milk is currently the basis for most IFs (Table 3).

IFs are based on the nutrient composition of human milk in order to provide adequate levels of macronutrients (carbohydrates, lipids, and proteins) and micronutrients (vitamins and minerals) to support growth [60,81]. Despite the similarities in MFGM proteome, the composition of bovine milk differs from human milk. An example is the content of essential unsaturated fatty acids, which is higher in human milk than in bovine milk because of the high rate of biohydrogenation processes of dietary unsaturated fatty acids in the rumen—α-linolenic acid shows a lower difference (0.5–2% and 1–2% of total milk FAs for bovine and human milk fat, respectively) than linoleic acid (1–3% and 8–18% of total milk FAs for bovine and human milk fat, respectively) [25,29,82,83]. In addition to nutrients, human breast milk also contains several bioactive compounds (Igs, enzymes, hormones) and live cells (e.g., leucocytes) that cannot be easily added to IFs [79,84]. All these elements prevent IFs from having the same composition as human breast milk, although the research in this field has advanced considerably.

In fact, several studies [49,57,60,77,85] have suggested that supplementing IFs with MFGM could provide beneficial effects because of the presence of bioactive compounds such as proteins and polar lipids in the MFGM, thus narrowing the gap between human breast milk and IFs. Many of these results were obtained through the supplementation of IF with the polar lipid fraction of the MFGM (phospholipids and sphingolipids), given that these compounds are the main contributors to the nutraceutical effects of the MFGM [6].

Few studies have investigated supplementing IF with MFGM proteins [78]. One hypothesis is that the protein content of IFs is usually higher than that of human milk, and this is likely due to the lower digestibility of cow milk proteins [77,86]. Further supplementation with proteins has thus rarely been taken into account, even though MFGM proteins have health-promoting effects such as preventing pathogen adhesion and infection [18,29].

In 2014, Billeaud and collaborators [87] evaluated the safety of two IFs supplemented with a lipid-rich or a protein-rich bovine MFGM fraction in healthy infants. The authors observed no considerable differences among the two supplemented formulas and the control (standard formula) in terms of weight gain, adverse events, and morbidity rates. They concluded that MFGM enrichment, both with lipids or proteins, could improve the level of similarity between breast milk and cow-based IFs [87]. In the same year, Timby and collaborators [77] evaluated the effect of a low-energy and low-protein formula supplemented with a protein-rich bovine MFGM fraction on healthy infants. Their results showed that the cognitive score (assessed with Bayley-III tests) was 4.0 points higher in the experimental formula group than in the standard formula group (105.8 ± 9.2 vs. 101.8 ± 8.0; *p* < 0.05), but was similar to the breastfed group (105.8 ± 9.2 vs. 106.4 ± 9.5; *p* > 0.05). This suggested that the experimental formula could decrease the gap in cognitive performance between breastfed and formula-fed infants [77].

Zavaleta and collaborators [88] evaluated the efficacy of a complementary food supplemented with a MFGM-enriched protein fraction on the health status of infants. They found that the supplementation improved infants’ health status by reducing episodes of diarrhea. Even though the authors did not use a supplemented IF but a supplemented complementary food, they achieved promising results, probably due to an amelioration of gut microbiota or positive changes in the developing immune system of the infants [88].

However, the relevance and the potential of MGFM is under investigation, not only as a source of several bioactive nutrients (fat-soluble vitamins including carotenoids and polar lipids) including MFGM proteins, but also for its role in fat digestion [89]. It was observed that bovine MFGM reduced the in vitro FA release from MFGs and this was probably due to the inhibitory effect of MFGM components and conformation on the pancreatic lipase activity [90]. Besides this, it is also important to mention that the composition of FAs in TG core of MFGs has a significant impact on the digestibility and the absorption of fat and other compounds, such as minerals. MCFAs are better absorbed than LCFAs, and therefore TGs made up mainly by MCFA result in being more digestible because they are better solubilized in the gut [91]. Moreover, calcium absorption is also higher in human subjects after the consumption of a MCFA-mixed meal compared to a LCFA-mixed meal [92]. In addition to these aspects, the positional distribution of FAs on the glycerol backbone is also crucial to determine FA absorption, whether as sn-2 monoglycerides or as free FAs (after the hydrolyzation by lipase of the sn-1 and sn-3 bounds). An example is palmitic acid (16:0)—in human milk it is found on the sn-2 glycerol position more than in bovine milk (>50% and 30–40%, respectively) [93]. The sn-2 position ensures higher absorption for palmitic acid than the sn-1 or sn-3 positions, also because free palmitic, being a LCFA, tends to form insoluble fatty soaps with calcium at intestinal pH conditions [94]. For this reason, the location of palmitic acid on the sn-2 position of glycerol ensures higher absorption for both the FA and the calcium, making human milk more digestible than cow-based IFs [91,95,96].

## 5. Conclusions

Milk has important nutritional features for newborns. Indeed, breast milk is a mixture of several bioactive compounds that modulate the GI tract and contribute to building the immune system of breast-fed infants. Moreover, breast milk is also important for brain development [81]. Bovine milk is the most used animal milk in the world [59] and it shares several features with other species’ milk, such as the particular occurrence of milk fat as MFGs surrounded by the MFGM. The lipids and proteins that constitute the MFGM supply it with many of the bioactive properties of milk [6]. Along with MFGM polar lipids, MFGM proteins have important health-promoting effects such as anti-adhesive and antimicrobial functions [18,29].

The growing interest in MFGM led researchers to study the MFGM proteins from a wider approach through proteomics. Proteomic methods have been performed to better clarify the role of MFGM proteins, leading to a deeper knowledge about them. Proteomics has the potential to enable the detection, identification, and characterization of proteins, as well as to analyze a large number of proteins simultaneously [97]. Comparative proteomic studies were performed to obtain information on the variations in MFGM proteome among different species [63]. There are variations in terms of protein expression level and molecular function across species, even though the major MFGM proteins are observed among all the species considered. The properties of the MFGM proteome of each species could be exploited to design products supplemented with MFGM fractions that meet specific needs, for example, the enhancement of the immune system, the regulation of cholesterol metabolism, or the supply of beneficial polar lipids to support cognitive function.

An example of the application of the MFGM proteome is found in the dairy industry, in particular in the supplementation of IFs [77,78,89]. The promising results obtained with the supplementation of IF with MFGM proteins [50,77] and polar lipids [85] underline once again the importance of MFGM in IF preparation—since cow milk-based IF is formulated to better resemble human breast milk, the MFGM supplementation could increase the presence of bioactive compounds in IF (usually at low levels in standard formula).

Future work is likely to be addressed towards a deeper comprehension of MFGM proteome and its variations across species and lactation stages. The overall aim is to further increase the knowledge of MFGM properties and to assess the potential of the supplementation of IFs with MFGM proteins.

## Figures and Tables

**Table 1:** Comparison of milk fat globule membrane (MFGM) content in different dairy products. Data are from Dewettinck et al., 2011, and Conway et al., 2014 [7,8].

Product	MFGM (mg/100 g)
Cheese (25% fat)	150
Milk (skimmed, 0.5% fat)	15
Milk (whole, 3.5% fat)	35
Yogurt (1.5% fat)	15
Cream (38% fat)	200

**Table 2:** Functions of the main components of bovine MFGM. Data from Lee et al., 2018 [29].

Components	Abbreviation	Functions
Polar Lipids		
Phosphatidylcholine	PC	Structural maintenance of MFGM; cholesterol regulation and lipoproteins metabolism
Phosphatidylethanolamine	PE	Structural membrane regulation
Phosphatidylinositol	PI	Cell signaling; PI3K-Akt pathway regulation
Phosphatidylserine	PS	Apoptosis regulation
Sphingomyelin	SM	Myelinization; metabolized to ceramide and sphingosine (second messengers that regulate cell growth and cell cycle)
Cholesterol	-	Structural maintenance of MFGM (lipid rafts complexes with SM)
Proteins		
Adipophilin	ADPH	Lipolysis regulation
Butyrophilin	BTN	MFG synthesis regulation
Mucin 1	MUC 1	Decoy receptor for pathogens; inhibition of in vitro rotavirus infectivity
Xanthine dehydrogenase/oxidase	XDH/XO	Structural maintenance of MFGM; antimicrobial activity (ROS/RNS production)
Fatty acid-binding protein	FABP	Fatty acid transport; MFG lipid synthesis
Breast related cancer antigens 1/2	BRCA 1/2	Onco-suppressor activity
Choline	-	Precursor of phospholipids and SM; hepatic lipid metabolism
Gangliosides	-	Cognitive development

**Table 3:** Comparison of human breast milk and cow-based infant formula composition (in terms of energy and macronutrients). Data are from the European Food Safety Authority (EFSA) Journal, 2014 [80].

Item	Human Breast Milk	Cow-Based IF
Energy (kcal/100 mL)	65	60–70
Digestible carbohydrates (g/100 kcal)	8.2–10.4	9–14
Lipids (g/100 kcal)	3.7–9.1	4.4–6
Proteins (g/100 kcal)	1.3 (0.8–2.1) ^a^	1.8–2.5

^a^ Mature human milk.

## Abbreviations

MFG	Milk fat globule
MFGM	Milk fat globule membrane
TG	Triglyceride
LDL	Low density lipoprotein
FA	Fatty acid
SCFA	Short-chain fatty acid
MCFA	Medium-chain fatty acid
LCFA	Long-chain fatty acid
PE	Phosphatidylethanolamine
PC	Phosphatidylcholine
PI	Phosphatidylinositol
PS	Phosphatidylserine
SM	Sphingomyelin
GI	Gastrointestinal
ADPH	Long-chain fatty acid
BTN	Butyrophilin
MUC1	Mucin 1
XDH/XO	Xanthine dehydrogenase/oxidase
PAS III	Periodic acid Schiff III
FABP	Fatty acid-binding protein
